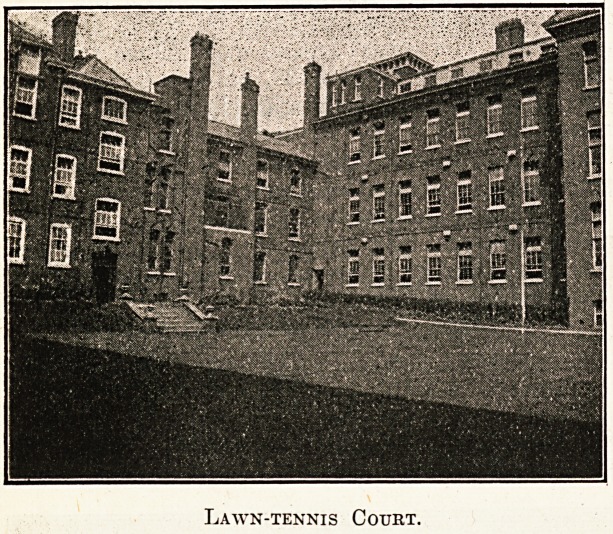# A Profitable Hospital Garden

**Published:** 1915-09-18

**Authors:** 


### A PROFITABLE HOSPITAL GARDEN.

**[The Editor invites Accounts with Photographs of other Hospital Gardens.]**

The Royal Devon and Exeter Hospital, Exeter, was founded in 1741, and is situated almost within the shadow of the towers of Exeter Cathedral, near the centre of the city. The gardens and grounds are immediately adjoining, and cover an area of about two acres. Half an acre is devoted to

The Garden.

recreation grounds, and comprises a tennis court, a croquet green, and a small lawn which is used by the patients.

With the exception of two greenhouses of about twenty feet each in length, which are used for growing cucumbers, tomatoes, etc., the whole of the garden is in the open, and no shelter of any kind is provided. The soil, on the whole, is kindly, so that ordinary kinds of vegetables do well. The fruit trees, of which there are a considerable number, especially apples and pears, are well-distributed and in good bearing condition. The arrangement of the garden for the various crops is excellent, due regard being had, in the rotation of crops, to suit the kind of soil to the liking of the particular crop assigned to it. Herein lies much of the success which follows such discrimination and forethought.

In passing through the gardens three notable features impress one and give the key to its profitableness; these are—method, orderliness, and the absence of weeds. Another feature is that nowhere is there any overcrowding, nor, on the other hand, any ground lying waste. In an old garden like this, naturally some of the fruit trees are sorts which, popular fifty years ago or more, have now been superseded by better; yet, the crops total up to a good average, and, to an establishment of this kind, come in very useful.

Apples, pears, plums, gooseberries, raspberries, strawberries and other fruits give an excellent successive supply according to the season. Vegetables do well. Apart from the fact of the garden being a source of profit from a financial point of view, the immense advantage to a hospital of being able to command a supply, within a few minutes, of cauliflowers, cabbages, broccoli, brussels sprouts, rhubarb, leeks, carrots, and suchlike in the morning for the same day's consumption cannot well be over-estimated. Garden peas, broad beans, runner beans, onions, beet, and lettuces crop splendidly in this garden, and the quality of all the vegetables is exceptionally high.

In former years the garden was leased to a market-grower, the tenant paying £25 per annum rent; but when repairs and other incidental expenses were deducted, and compensation allowed for a small piece of ground taken out of the garden during the tenancy, the net income reached vanishing-point. At that time, too, from £20 to £25 per annum was paid for the care of the hospital grounds and a small piece of kitchen-garden.

The hospital does not debit any rent for the garden, the annual rates and taxes on which average about £5. The actual annual expenditure for the last four years averaged £128, but this included an item of £22 for repairing and painting the greenhouses in 1912. The expenditure for 1914 was £115. This amount includes the wages of the two gardeners (a man and a strong youth).

Since the committee took the management of the garden into its own hands, the income, year by year, has steadily increased, as the following four years' returns will show:—

| | | | |
|---|---|---|---|
| **Garden produce in 1911** | **yielded** | **£144** |
| **"** | **1912** | **"** | **147** |
| **"** | **1913** | **"** | **157** |
| **"** | **1914** | **"** | **177** |

and the returns for the present year promise to maintain or exceed the gradual increase in revenue.

Patients' Recreation Ground.

It may be mentioned that the cropping of the garden permits of the cultivation of many flowers for picking for the wards, such as sweet peas, asters, stocks, mignonette, and other useful annuals for cutting. In addition to these, Mr. P. C. M. Veitch has for some years made a liberal present of bulbs—hyacinths, tulips, daffodils, etc.—for planting in the borders and odd corners of the grounds.

The yield of crops has been very satisfactory, and, compared with the outlay on seeds, etc., shows a handsome profit, as the following tables will show. The entire cost in renewing worn-out tools last year was £2 18s.; for apple trees and gooseberry bushes, £1 7s.; and for manure (12 loads), £3 12s. It should, however, be mentioned that several loads of manure were sent as a present from governors of the hospital, who take a special interest in the garden.

The extracts here given deal with one year's work only—the year 1914. In purchasing the requirements for the garden in the way of seeds, potatoes, tools, fruit trees and bushes, etc., no tender is asked for, the only stipulation made being that in every case the best quality be supplied. The tradesmen, in consideration of the nature of the institution, charge reduced prices where possible. The selection of the sorts to be grown, to a great extent, is left to

Lawn-tennis Court.

Mr. Veitch, who is a governor of the institution and personally superintends the whole of the practical work of the garden.

In 1914 the gardeners' day-book showed the following aggregate yields. In the case of vegetables, the quantity of seed sown is given which produced the crop.

Fruit.

| | |
|---|---|
| 19,505 Apples | 7¾ cwt. Raspberries |
| 4,862 Pears | 5¼ cwt. Strawberries |
| 253 Plums | 1¾ cwt. Gooseberries |
| 34 Peaches | 32 lbs. Red Currants |
| 96 lbs. Mulberries | 8 lbs. Black Currants |

Vegetables.

| | Seed Sown | Yield |
|---|---|---|
| Potatoes ... ... | 5 cwt. | 45 cwt. |
| Peas ... ... ... | 7 qts. | 67 pecks |
| Beans (broad) ... ... | 2 qts. | 20 pecks |
| Beans (runner) ... ... | 3 qts. | 567 lbs. |
| Beet ... ... ... | 2 ozs. | 5½ cwt. |
| Borecole (greens) ... | 4 ozs. | 42 bags |
| Broccoli ... ... | 1½ oz. | 106 heads |
| Brussels sprouts ... | 1 oz. | 253 lbs. |
| Cabbages ... ... | 4½ ozs. | 8,685 heads |
| Cauliflowers ... ... | 1½ oz. | 386 heads |
| Carrots ... ... ... | 2 ozs. | 1½ cwt. |
| Celery ... ... ... | Packet (6d.) | 138 heads |
| Cucumbers ... ... | 2 pkts. (2s. 6d.) | 94 fruits |
| Leeks ... ... ... | 1 oz. | 140 dozen |
| Lettuces ... ... | 2 ozs. | 786 heads |
| Onions ... ... ... | 8 ozs. | 4½ cwt. |
| Parsley ... ... ... | 1 oz. | 15s. worth |
| Parsnips ... ... ... | 2 ozs. | 3¾ cwt. |
| Radishes ... ... ... | 4 ozs. | 11 lbs. |
| Tomatoes ... ... | Packet (1s. 6d.) | 3¼ cwt. |
| Turnips ... ... ... | 1 pint | 3½ cwt. |
| Vegetable marrows ... | Packet (6d.) | 147 fruits |

In addition to the above, the returns show 1,115 heads of asparagus, 30 lbs. of seakale, 8 cwt. of rhubarb, and sundry other vegetables and herbs.

## Figures and Tables

**Figure f1:**
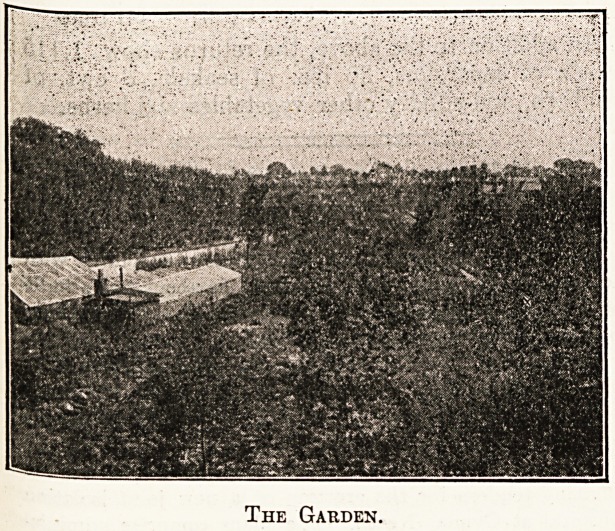


**Figure f2:**
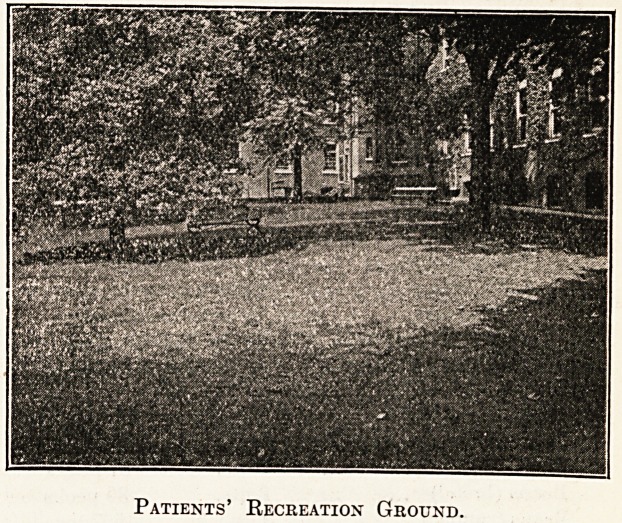


**Figure f3:**